# Elimination of Pharmaceutical Compounds from Aqueous Solution through Novel Functionalized Pitch-Based Porous Adsorbents: Kinetic, Isotherm, Thermodynamic Studies and Mechanism Analysis

**DOI:** 10.3390/molecules29020463

**Published:** 2024-01-17

**Authors:** Qilin Yang, Hongwei Zhao, Qi Peng, Guang Chen, Jiali Liu, Xinxiu Cao, Shaohui Xiong, Gen Li, Qingquan Liu

**Affiliations:** 1 School of Material Science and Engineering, Hunan University of Science and Technology, Xiangtan 411201, China; a3346529088@163.com (Q.Y.); pengqi408@126.com (Q.P.); cghnust@163.com (G.C.); 17373487964@163.com (J.L.); xxcao@hnust.edu.cn (X.C.); xiongshaohui@hnust.edu.cn (S.X.); genkevinli@hnust.edu.cn (G.L.); 2 Hunan Provincial Key Laboratory of Advanced Materials for New Energy Storage and Conversion, Hunan University of Science and Technology, Xiangtan 411201, China

**Keywords:** HCP adsorbents, pitch-based, magnetic, PPCPs

## Abstract

The long-term presence of PPCPs in the aqueous environment poses a potentially significant threat to human life and physical health and the safety of the water environment. In our previous work, we investigated low-cost pitch-based HCP adsorbents with an excellent adsorption capacity and magnetic responsiveness through a simple one-step Friedel–Crafts reaction. In this work, we further investigated the adsorption behavior of the prepared pitch-based adsorbents onto three PPCP molecules (DFS, AMP, and antipyrine) in detail. The maximum adsorption capacity of P-MPHCP for DFS was 444.93 mg g^−1^. The adsorption equilibrium and kinetic processes were well described through the Langmuir model and the proposed secondary kinetic model. The negative changes in Gibbs free energy and enthalpy reflected that the adsorption of HCPs onto PPCPs was a spontaneous exothermic process. The recoverability results showed that the adsorption of MPHCP and P-MPHCP onto DFS remained above 95% after 10 adsorption–desorption cycles. The present work further demonstrates that these pitch-based adsorbents can be used for multiple applications, which have a very extensive practical application prospect.

## 1. Introduction

Global economic and population growth is currently leading to the troubling reality of the pervasive contamination of freshwater ecosystems. Pharmaceuticals and personal care products (PPCPs), along with other trace organic pollutants, have become pervasive sources of pollution [1,2]. PPCPs are available in a wide range of categories including antibiotics, anti-inflammatory drugs, β-blockers, anticonvulsants, lipid modulators, etc. [3,4]. According to World Health Organization (WHO) statistics, the global consumption of pharmaceuticals has been estimated at 100,000 tons per year [5]. PPCPs can re-enter the environment through various pathways [6,7,8,9]. Despite undergoing conventional wastewater treatment, significant PPCP residues can persist in effluent, with certain pharmaceuticals like diclofenac exhibiting removal rates as low as 10% [10,11,12]. Alarmingly, these remnants of novel antibiotic products can contribute to the development of drug-resistant bacteria, thereby escalating public health concerns.

Traditional wastewater treatment methods like activated sludge, membrane filtration, and photodegradation suffer from inherent shortcomings, notably secondary pollution, high costs, and limited efficiency [13,14,15,16]. For instance, the cost of biological treatment ranges from 0.17 to 0.53 €/m^3^, but it achieves only a 55% removal rate for PPCPs. Photocatalysis comes at a steep cost of 10.36 €/m^3^, but it does achieve high removal rates. Hybrid technology, with a cost of 0.17–0.75 $/m^3^, may necessitate pre-treatment for specific wastewater samples; however, the integration process demands more processing units, leading to increased energy consumption [17]. As an alternative, adsorption has proven to be effective as it is simple to operate, less likely to cause secondary pollution, and the adsorbent can be regenerated. However, traditional adsorption materials have restricted pores and poor chemical stability [18]. To address this challenge, organic porous materials are being developed as new, effective adsorbent materials to meet current demands.

Porous organic polymers (POPs) represent a promising breakthrough in adsorbent materials for the removal of PPCPs from wastewater [19,20]. These materials are crafted by covalently linking organic monomers, thereby resulting in a customizable network structure featuring high specific surface areas, exceptional chemical and thermal stability, and a uniform pore configuration [21]. Hypercrosslinked polymers have displayed remarkable efficiency in the elimination of waterborne contaminants including PPCPs. Ravi et al. synthesized two new phosphate-based POPs and evaluated their effectiveness in adsorbing PPCPs [22]. The results showed that the maximum adsorption of P-POP-2 onto caffeine, diclofenac, and carbamazepine was 301, 217, and 248 mg g^−1^, respectively. Gan et al. pioneered the creation of oxygen-rich hypercrosslinked polymers through a tandem of continuous Friedel–Crafts reactions [23]. This innovative approach significantly enhanced the adsorption of aniline onto these synthesized polymers, showcasing a remarkable maximum capacity of 178.4 mg g^−1^ at 303 K. Hao et al. successfully synthesized a covalent organic framework (COF-SO_3_H) that was functionalized with sulfonic acid (-SO_3_H) groups for the effective removal of PPCPs [24]. It was shown that COF-SO_3_H exhibited a strong affinity for all 13 PPCPs studied, with especially noteworthy properties in the adsorption of diclofenac. COF-SO_3_H achieved a maximum adsorption capacity of 770 mg g^−1^. Organic porous polymers hold immense promise for PPCPs adsorption applications. Hence, the pursuit of cost-effective, highly efficient, and readily producible POP adsorbents carries substantial practical importance.

Pitch poses potential hazards due to its composition containing pyridine, benzene, and naphthalene, which can lead to severe harm through skin contact, inhalation, or extended environmental exposure. Consequently, the environmentally responsible use of pitch, particularly its transformation into an adsorbent for water treatment, represents a highly desirable application [25]. Yang and collaborators have showcased the remarkable versatility of pitch-based hypercrosslinked polymers (PHCPs) as carbon precursors for crafting nitrogen-doped layered porous carbon materials (N-PHCP-900), notably enhancing oxygen reduction reaction (ORR) performance. N-PHCP-900 exhibited exceptional ORR performance in alkaline electrolytes, outperforming both commercial Pt/C and most non-precious metal electrocatalysts [26]. This demonstrates that the removal of PPCPs through hyper-crosslinked modified pitch-based polymers is a feasible approach, especially considering the cost-effectiveness and the simple procedure involved [27,28,29].

**Figure 1:**
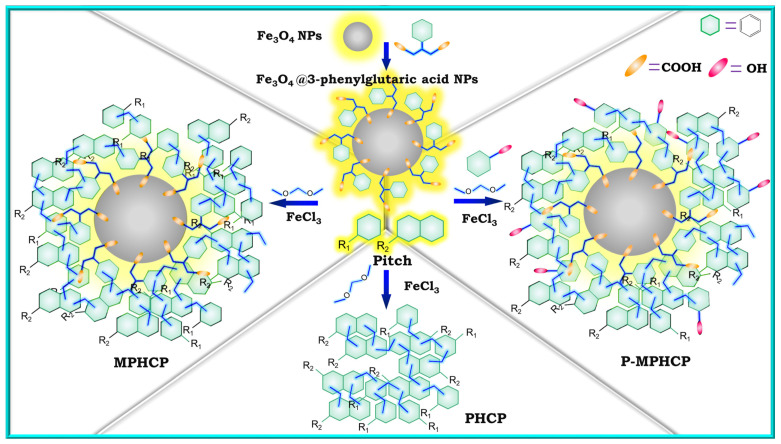
The synthetic scheme of Fe_3_O_4_@3-phenylglutaric acid NPs, PHCP, MPHCP, and P-MPHCP.

In our previous work, we designed and synthesized functionalized Fe_3_O_4_ magnetic nanoparticles (Fe_3_O_4_@3-phenylglutaric acid NPs) to both magnetize and enhance the functionality of pitch-based hypercrosslinked polymers. Meanwhile, by utilizing Friedel–Crafts reaction method, three cost-effective, pitch-based, high-performance adsorbents (PHCP, MPHCP, and P-MPHCP) were prepared [30]. As shown in Figure 1, pure pitch was used to synthesize PHCP. Through the combination of pitch and Fe_3_O_4_@3-phenylglutaric acid NPs, MPHCP, a magnetic pitch-based HCP featuring carboxyl functionalization, was synthesized. To enable a more precise comparison of the effects of the two different functional groups, P-MPHCP was synthesized using pitch, Fe_3_O_4_@3-phenylglutaric acid NPs, and phenol. This was undertaken while considering the intricate composition of pitch with the potential presence of phenolic hydroxyl groups. The isotherms, kinetics, thermodynamics, and recoverability of these pitch-based HCPs for the adsorption of three PPCPs (diclofenac (DFS), 4-acetylaminophenol (AMP), and antipyrine) were investigated in detail. The mechanism of PPCP adsorption was elucidated through infrared spectra and XPS analysis of the samples before and after adsorption.

## 2. Results and Discussions

### 2.1. pH-Dependent Adsorption Behaviors of Pitch-Based HCPs

**Figure 2:**
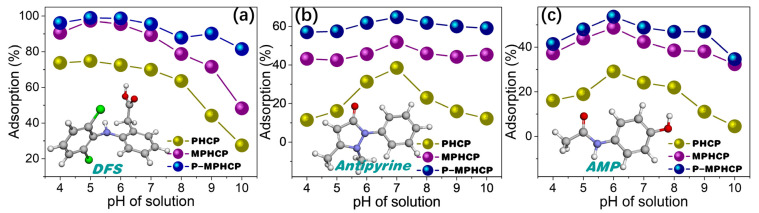
pH-dependent adsorption capacities of the pitch-based HCP adsorbents to DFS (**a**), antipyrine (**b**), and AMP (**c**).

There is a statistically significant effect of the pH level on the adsorption process, mainly in terms of the surface charge and morphology of the adsorbent [31]. Exploring the optimal pH conditions for the adsorption of various PPCP yields crucial insights. These insights are essential for designing efficient and environmentally friendly treatment methods for wastewater containing these contaminants. The equilibrium concentrations of the samples were assessed with a standard curve, and the linear Beer–Lambert relationship was given through the calibration plot of absorbance versus the concentration, as shown in Figure S1. Figure 2 demonstrates the effect of pitch-based HCPs on the adsorption efficiency (%) of three PPCPs (DFS, AMP, and antipyrine) at different pH levels (4~10). The results showed a significant increase in the adsorption efficiency with an increasing pH level, reaching a maximum level of efficiency at the pH levels of 5, 6, and 7 for DFS, AMP, and antipyrine, respectively. In the case of a relatively low pH level (<4), the lower adsorption rate of the adsorbents is attributed to the fact that the protonated forms of all PPCPs may limit interaction with the surface of the protonated adsorbent. With a higher pH level, the deprotonated forms of PPCPs may weaken interaction with the surface sites of the adsorbent due to the increase in the concentration of free hydroxyl ions in the aqueous solution, thereby leading to a decrease in the adsorption performance [32]. Therefore, further adsorption studies were carried out at the optimal pH levels of 5, 6, and 7 for DFS, AMP, and antipyrine, respectively.

### 2.2. Adsorption Kinetics

**Figure 3:**
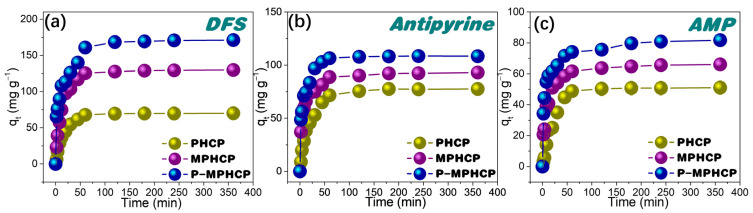
Adsorption kinetic results for pitch-based HCP adsorbents onto DFS (**a**), antipyrine (**b**), and AMP (**c**).

To understand the adsorption rate, the effect of the contact time of pitch-based HCPs onto PPCPs at the optimal pH level on their adsorption performance was investigated. As shown in Figure 3, the adsorption process of PPCPs through the adsorbent first exhibited a rapid increase and then tended to an adsorption equilibrium of around 80 min. Among the three adsorbents, P-MPHCP showed the strongest adsorption capacity for PPCPs; the order of adsorption capacity was P-MPHCP > MPHCP > PHCP, and the adsorption efficiencies of P-MPHCP for DFS, AMP, and antipyrine were 85.58, 40.88, and 54.21%, respectively. In addition, P-MPHCP, MPHCP, and PHCP all showed more a prominent adsorption capacity for DFS, with adsorption efficiencies of 85.58, 64.92, and 35.02%, respectively. The specific surface areas of PHCP, MPHCP, and P-MPHCP in BET tests were 531, 483, and 468 m^2^ g^−1^, respectively. The introduction of functional groups, such as carboxyl or phenolic hydroxyl groups, plays a crucial role in enhancing the adsorption capacity of MPHCP and P-MPHCP for PPCPs. This leads to PHCPs having a larger specific surface area but a lower adsorption capacity. Consequently, it can be deduced that the incorporation of functional groups, such as carboxyl or phenolic hydroxyl, significantly improves the adsorption capacity of adsorbents onto PPCPs.

For a more comprehensive assessment of the adsorption kinetics, the adsorption data were subjected to a thorough analysis using pseudo-first-order and pseudo-second-order kinetic models [33,34], as described by Equations (1) and (2), respectively.

(1) $$\mathrm{Pseudo}-\mathrm{first}\;\mathrm{order}:\;\ln\;\left(q_{e}-q_{t}\right)=\ln\;q_{e}-K_{1}t$$

(2) $$\mathrm{Pseudo}-\mathrm{second}\;\mathrm{order}:\;\frac{t}{q_{t}}=\frac{t}{q_{e}}+\frac{1}{q_{e}^{2}\;K_{2}}$$

**Figure 4:**
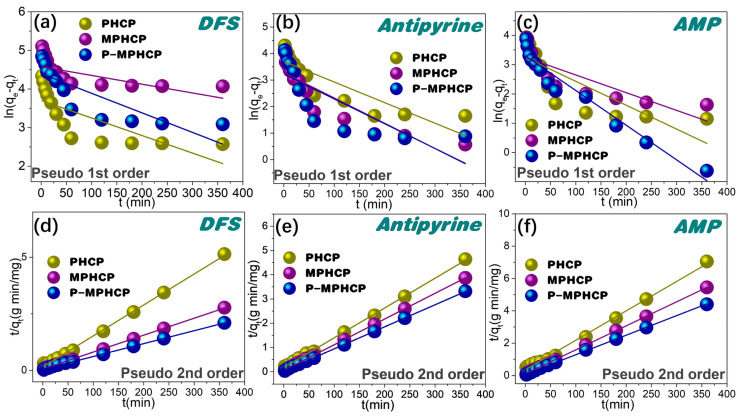
Adsorption kinetics for pitch-based HCP adsorbents onto PPCPs fitted with the pseudo-first order kinetic models of (**a**–**c**) and the pseudo-second order kinetic models of (**d**–**f**).

Herein, *q_t_* (mg g^−1^) represents the adsorption capacity at time *t* (minutes), while *q_e_* (mg g^−1^) represents the adsorption capacity at equilibrium. The parameters *K*_1_ (min^−1^) and *K*_2_ (g mg^−1^ min^−1^) stand for the rate constants associated with pseudo-first-order and pseudo-second-order adsorption, respectively. The proposed first-order and proposed second-order adsorption kinetics of HCPs on PPCPs are shown in Figure 4, and the parameter fitting results are shown in Table 1. In contrast, the pseudo-second order kinetic model, as depicted in Figure 4 and detailed in Table 1, exhibits an exceptionally precise fit for the PHCPs, yielding *R*^2^ values ranging from 0.9959 to 0.9999. The results demonstrated that the fitted results of the pseudo-second-order kinetic model were greatly superior to the pseudo-first-order kinetic model. P-MPHCP and MPHCP exhibit significantly higher adsorption capacities for PPCPs compared with PHCP. This is primarily due to the increased availability of adsorption sites facilitated through the carboxyl/phenolic hydroxyl groups. As a result, the adsorption mechanism governing the interaction between HCPs and PPCPs was primarily chemisorption-based, with the functional groups contributing to the augmentation of the available adsorption sites.

The potential for intraparticle diffusion was investigated through the utilization of the Weber–Morris model, represented through the following Equation:

(3) $$q_{t}=k_{IP}t^{0.5}+C$$

**Figure 5:**
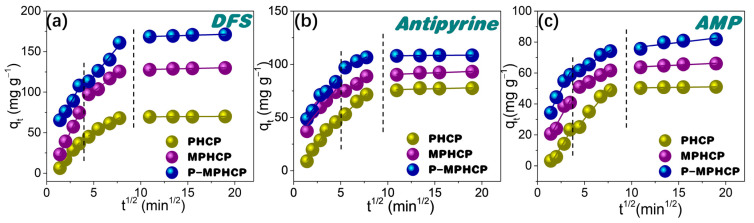
Weber–Morris intraparticle diffusion model for PHCP, MPHCP, and P-MPHCP toward DFS (**a**), antipyrine (**b**), and AMP (**c**) at initial concentrations of 200 mg L^−1^.

In this equation, *k_IP_* represents the intraparticle diffusion rate constant (mg g^−1^ min^0.5^), while *C* is the intercept resulting from the plot of *q_t_* against *t*_0.5_. This plot should yield a straight line with a slope equal to *k_IP_* when the adsorption mechanism adheres to the intraparticle diffusion process (as shown in Figure 5). The intraparticle diffusion model for PPCPs can be divided into three distinct steps as follows: (1) an immediate ascension phase driven by interactions between PPCPs and the functional groups; (2) the process of intraparticle diffusion within the porous structure; and (3) reaching an equilibrium stage [35]. It is worth highlighting that the linear fitting plots did not start from the point of origin, suggesting that intraparticle diffusion is not the sole limiting factor of the rate [36]. The effectiveness of the adsorption of PPCPs might be influenced by a combination of factors including the size of the PPCP molecules and the distribution of the adsorbent’s pore structure.

### 2.3. Effect of Initial Concentration

**Figure 6:**
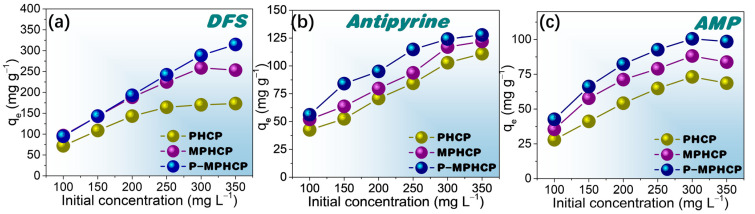
Isotherm adsorption capacity for pitch-based HCP adsorbents onto DFS (**a**), antipyrine (**b**), and AMP (**c**) with different initial concentrations at 25 °C.

To investigate the effect of the initial concentration on the adsorption capacity of the adsorbents, isothermal equilibrium adsorption experiments were conducted at the optimal pH solution. These experiments covered a range of initial PPCP concentrations from 100 to 350 mg L^−1^. Figure 6, Figures S2 and S3, depict the influence of the initial PPCP concentration on the adsorption capacity at temperatures of 25, 35, and 45 °C, respectively. Comparing the adsorption properties of the three pitch-based adsorbents onto PPCPs, P-MPHCP showed the most exceptional adsorption capacity. This was followed by MPHCP and finally PHCP, which was neither magnetized nor modified and showed the weakest adsorption capacity. For instance, the adsorption efficiency of DFS in solution was significantly high for all pitch-based HCPs (≥94%) at concentrations ranging from 100 to 200 mg L^−1^. At lower solution concentrations, the introduction of phenolic hydroxyl groups did not significantly affect the adsorption. This could be attributed to the availability of more adsorption sites than needed. When the initial concentration was set at 350 mg L^−1^, the maximum adsorption capacities for DFS onto PHCP, MPHCP, and P-MPHCP were found to be 173.20, 253.38, and 316.78 mg g^−1^, respectively. It is indicated that the introduction of the carboxyl and phenolic hydroxyl groups after magnetic functionalization does indeed enhance the adsorption capacity of the adsorbent onto PPCPs. Moreover, all three adsorbents showed a consistent trend in the adsorption capacity to PPCPs along the order of DFS > antipyrine > AMP.

### 2.4. Adsorption Isotherms

The adsorption efficiency of the adsorbents was investigated using adsorption isotherms to evaluate the adsorption of the adsorbents, the correlation between equilibrium adsorption and equilibrium concentration, and the mutual interaction between the adsorbent and the adsorbent surface. Equations (4)–(6) for the Freundlich, Langmuir, and Dubinin–Radushkevich (*D*–*R*) models are represented as follows [37,38,39]:

(4) $$\mathrm{Langmuir}:\;\frac{C_{e}}{q_{e}}=\frac{C_{e}}{q_{max}}+\frac{1}{\;q_{\max\;}K_{L}}$$

(5) $$\mathrm{Freundlich}:\;\ln\;q_{e}=\ln K+\frac{\ln C_{e}}{n}$$

(6) $$\mathrm{Dubinin}–\mathrm{Radushkevich}\;(D–R):\;\ln\;q_{e}=\ln q_{D-R}-B\varepsilon^{2},\;\varepsilon =RTln\left(1+\frac{1}{C_{e}}\right),\;E_{a}=\frac{1}{\sqrt{2B}}$$

In these equations, *q_e_* represents the adsorption capacity (mg g^−1^), *C_e_* is the equilibrium concentration (mg L^−1^), *q_max_* stands for the maximum adsorption capacity (mg g^−1^), *K_L_* is the binding strength constant (L mg^−1^), while *K* and *n* are constants denoting adsorption capacity and intensity. *B* is the adsorption energy constant (mol^2^ kJ^−2^), *ε* signifies the Polanyi potential, *R* represents the gas constant (8.314 J (mol K)^−1^), T indicates the absolute temperature (*K*), and *E_a_* represents the mean energy of adsorption (kJ mol^−1^).

**Figure 7:**
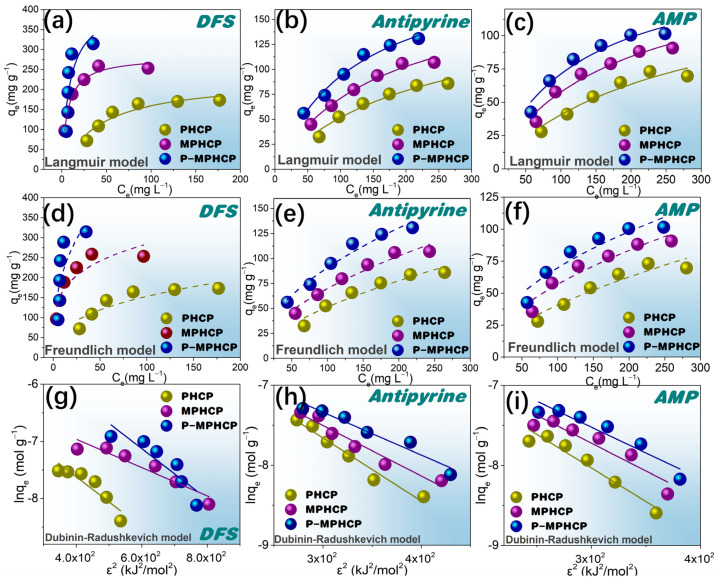
Langmuir (**a**–**c**), Freundlich (**d**–**f**), and D–R (**g**–**i**) isotherms for the adsorption of pitch-based HCP adsorbents onto DFS, antipyrine, and AMP at 25 °C.

Freundlich-, Langmuir-, and D–R-fitted isotherms for the adsorption of PPCPs at 25 °C are shown in Figure 7a–i. Two fitted models for 35 and 45 °C are shown in Figures S4 and S5. The model Langmuir and Freundlich model fitting parameters are shown in Table 2; for almost all isotherms, the Langmuir model is more suitable than the Freundlich model. Therefore, the adsorption behavior of pitch-based HCPs onto PPCPs is analogous to that of monolayer adsorption. In addition, the Langmuir isotherm fit from Table 2 shows that the *R_L_* values are between 0 and 1, indicating that the adsorption process is spontaneous [40]. The parameters obtained from the Freundlich isotherms in Table 2 show that the values of 1/*n* are in the range of 0.1~0.5, indicating that the pitch-based HCPs have good adsorption performance on PPCPs. Based on the Langmuir model, the P-MPHCP’s monolayer adsorption capacities were 444.93, 160.80, and 211.12 mg g^−1^ for DFS, AMP, and antipyrine, respectively. The Langmuir model assumes a uniform surface with consistent activation energy for adsorption, making it suitable for describing adsorption on homogeneous surfaces. On the other hand, the Freundlich model is better suited for surfaces that exhibit a high degree of heterogeneity. Analyzing the results presented in Table 2, Tables S1 and S2, it becomes evident that the Langmuir model provides a more accurate fit compared with the other two isotherm models. This suggests that the adsorption process likely involves predominantly monolayer adsorption, primarily driven through a site-to-site adsorption mechanism. Additionally, the *E_a_* values obtained from the D–R isotherm model offer insights into the type of adsorption. The calculated *E_a_* values fall within the range of 8 to 16 kJ mol^−1^, indicating that the adsorption is primarily characterized by chemisorption.

### 2.5. Adsorption Thermodynamics

The adsorption isotherm is affected by temperature, and the van’t Hoff equation can be used to define the thermodynamic parameters. The effect of temperature is illustrated through the adsorption of PPCPs at 25, 35, and 45 °C, as shown in Table 3. The data indicate that temperature is detrimental to adsorption, and the higher the temperature the smaller the *q*_e_. Moreover, these results were fitted with the Langmuir and Freundlich models, as shown in Figures S4 and S5, and the fitted parameters are listed in Tables S1 and S2. The thermodynamic values of adsorption such as the enthalpy of adsorption ∆*H* (kJ mol^−1^), free energy of adsorption ∆*G* (kJ mol^−1^), and entropy of adsorption ∆*S* (J (mol K) ^−1^) can be calculated through the following Equations (7)–(9) [41,42,43]:

(7) $$\ln K_{L}=-\frac{\Delta H\;}{RT}+\ln K_{0}$$

(8) $$\Delta G=-\mathrm{RT}\ln K_{L}$$

(9) $$\Delta S=\frac{\Delta H-\Delta G}{T}$$

Here, *K_L_* represents the adsorption equilibrium constant with units of L mol ^−1^, *R* is the universal gas constant at 8.314 J (mol K) ^−1^, *T* signifies the absolute temperature in Kelvin (K), and *K*_0_ is a constant.

Table 3 reveals that the ∆*H* value is negative, indicating an exothermic adsorption process of surface HCPs onto PPCPs. The thermodynamic parameters, as derived from Equations (9)–(11), are presented in Table 3. Notably, all the ∆*G* values are observed to be negative across all temperatures during the PPCP adsorption process. This observation indicates that the removal of PPCPs is a spontaneous and thermodynamically favorable process. Furthermore, it is observed that as the operating temperature increases the value of ∆*G* also increases. This suggests that higher operating temperatures do not promote an enhancement in adsorption capacity. In addition, the negative values of ∆*H* indicate that the adsorption of PPCPs is indeed an exothermic reaction.

### 2.6. Recyclability of Pitch-Based HCPs

**Figure 8:**
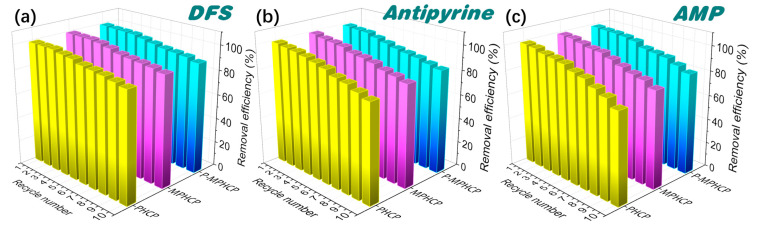
Cycled adsorption/desorption runs of PHCP, MPHCP, and P-MPHCP for DFS (**a**), antipyrine (**b**), and AMP (**c**).

The regeneration of pitch-based HCPs was evaluated to verify their recoverability and stability. Adsorption–regeneration cycle experiments were performed through efficient separation with an applied magnetic field, and the experimental conditions of adsorption were kept constant during each cycle. Figure 8 shows ten adsorption/desorption cycle experiments of pitch-based HCPs onto PPCPs. The experimental results showed that the adsorption efficiency of absorbents decreased slightly (<5%) after the first five repeated cycles. Finally, it was observed that the adsorption efficiency of MPHCP was 88.4, 74.01, and 80.05% for DFS, AMP, and antipyrine, respectively; the adsorption efficiency of P-MPHCP was 90.9, 82.19, and 84.54% for DFS, AMP, and antipyrine, respectively. Hence, the prepared pitch-based HCP adsorbents exhibit excellent reusability, high adsorption capacity, ease of separation, and hold significant promise for efficiently removing PPCPs from wastewater.

### 2.7. Adsorption Mechanism

**Figure 9:**
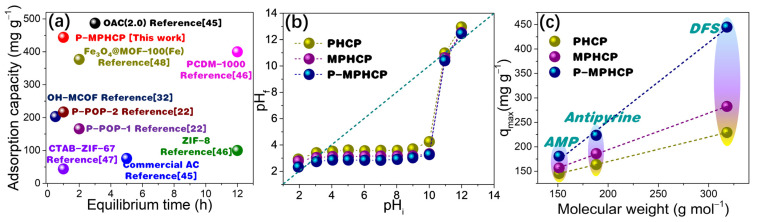
Comparisons of equilibrium time vs. adsorption capacity for PPCP adsorption in P-MPHCP and previously reported benchmark materials (**a**) [22,32,45,46,47,48]; determination of the pHpzc of pitch-based HCP adsorbents through the pH drift method (**b**); adsorption quantity relationship with the molecular weight of PPCPs (**c**).

As shown in Figure 9a, P-MPHCP demonstrated a notably higher adsorption capacity when compared with the data provided in Table S3, highlighting its superior performance. Understanding the adsorption mechanism holds paramount importance, serving to not only clarify the fundamental principles of adsorption but also to pave the way for potential commercial applications.

#### 2.7.1. Pore Structure

While highly porous adsorbents with extensive specific surface areas are typically expected to enhance adsorption capacity, our experiments revealed an interesting outcome. Despite possessing a highly specific surface area, PHCP exhibited a relatively lower adsorption capacity compared with P-MPHCP. This suggests that factors beyond specific surface area play a significant role in pharmaceutical compound adsorption. Specialized interactions between the adsorbent and pharmaceutical compounds are particularly noteworthy for influencing the adsorption process [44].

#### 2.7.2. Electrostatic Interaction

Electrostatic interactions between adsorbate molecules and adsorbent surfaces can occur due to the presence of ionizable functional groups [49]. The solution’s pH level plays a crucial role as it impacts both the surface charge of the adsorbent and the extent of PPCP dissociation [50]. As shown in Figure 9b, the points of zero charge (pH_pzc_) for pitch-based HCP adsorbents measured through the pH drift method are 3.46, 3.06, and 2.53, respectively. Thus, the surface of the pitch-based absorbents was negatively charged (pH > pH_pzc_) when adsorption studies were performed at the optimal pH levels of 5, 7, and 6 for DFS, AMP, and antipyrine, respectively. For pharmaceutical molecules, at pH > pK_a_, the pharmaceutical molecules are negatively charged and at pH < pK_a_, the pharmaceutical molecules are in a neutral form [51]. Hence, under optimal pH conditions, both the pitch-based adsorbents and DFS (with a pKa of 4.15) exhibit negative charges, whereas antipyrine (with a pK_a_ of 1.4) also carries a negative charge, and AMP (with a pK_a_ of 9.38) remains neutral (Figure S6) [52,53]. Consequently, it can be inferred that electrostatic interactions do not play a dominant role in the adsorption of PPCPs onto pitch-based adsorbents.

#### 2.7.3. Molecular Dimension

As displayed in Figure 9c the molecular weight of PPCPs had a strong positive correlation with the gravimetric capture capacity (mg g^−1^) of pitch-based adsorbents. A consistent relationship between the molecular weight of pharmaceutical compounds and their adsorption capacities onto porous polymer adsorbents was observed for pitch-based adsorbents [22]. Notably, larger molecules such as DFS exhibited higher qm values compared with smaller molecules like AMP. This indicates that the number of adsorption sites distributed in the pores of the pitch-based adsorbent should be approximately identical. This is particularly relevant in the case of adsorption sites fully occupied by several PPCP molecules after adsorption saturation [54]. Meanwhile, the adsorption interaction mechanism of the pitch-based adsorbent for several PPCP molecules should be analogous, and consequently the adsorption capacity exhibits a high degree of correlation with the molecular weight. Therefore, for the three PPCPs, it is possible that some co-occurring adsorption interactions, such as the formation of the hydrogen-bonding interaction as well as the π-π* dispersion interaction, may play a major contribution in the adsorption process.

#### 2.7.4. Hydrogen-Bonding Interaction

**Figure 10:**
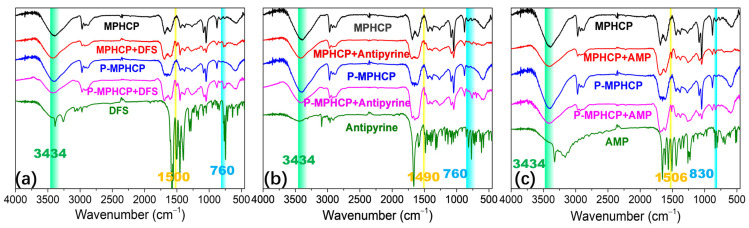
FTIR spectra of adsorbents before and after DFS (**a**), antipyrine (**b**), and AMP (**c**) adsorption.

The hydroxyl groups on the surface of the pitch-based adsorbent act as hydrogen donors. In turn, the oxygen or nitrogen atoms in the PPCPs serve as hydrogen acceptors, resulting in a dipole–dipole hydrogen-bonding interaction. Another type of hydrogen bonding, known as Yoshida-type, occurs between the hydroxyl groups of adsorbents and the aromatic rings of the PPCPs [55]. As depicted in Figure 10, similar stretching bands were observed at 760, 830, and 1500 cm^−1^ for both PPCPs and PPCP-adsorbed MPHCPs, confirming the presence of adsorbed PPCPs in pitch-based adsorbents. It becomes evident that following the adsorption process, the band corresponding to hydroxyl groups (at 3434 cm^−1^) exhibited a reduced intensity. This reduction confirms the presence of hydrogen-bonding interactions between the PPCPs and the pitch-based adsorbents. Notably, the decrease in intensity was more pronounced for P-MPHCP compared with MPHCP, possibly due to OH interactions [56].

**Figure 11:**
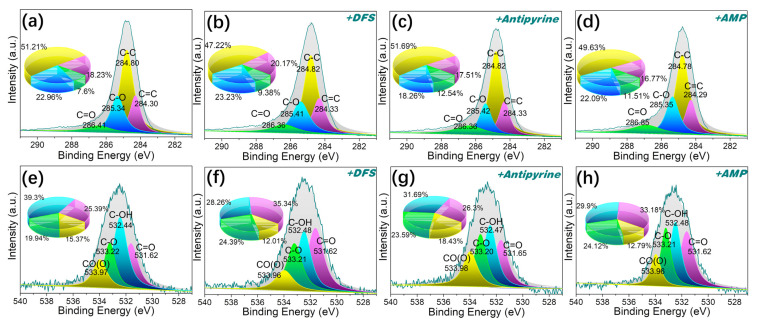
The XPS spectra of C1s (**a**–**d**) and O1s (**e**–**h**) for MPHCP before and after PPCP adsorption.

As shown in Figure 11e–h, analysis of the O1s spectrum was deconvoluted into four peaks for MPHCP corresponding to C=O (531.62 eV), C–OH (532.44 eV), C–O (533.21 eV), and C(O)O (533.96 eV) [57]. After adsorption, there was a notable reduction in the atomic fractions of C-OH in the O1s spectra of MPHCP, amounting to a decrease of approximately 9~11%. This observation suggests that dipole–dipole hydrogen bonding played a significant role in the adsorption of PPCPs. For P-MPHCP, after the introduction of a significant number of phenolic groups, the positions and atomic fractions of several oxygen atoms before and after adsorption were approximately unchanged, which was mainly attributable to Yahida-type H bonding with the benzene ring.

#### 2.7.5. π-π* Dispersion Interaction

**Figure 12:**
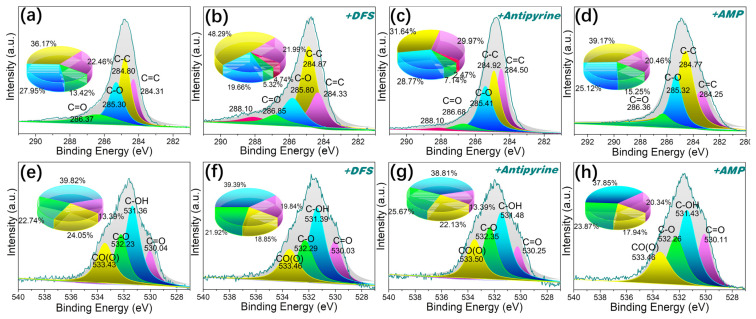
The XPS spectra of C1s (**a**–**d**) and O1s (**e**–**h**) for P-MPHCP before and after PPCP adsorption.

Given the abundance of aromatic structures in pitches, it is plausible that π-π* dispersion interactions between these aromatics in the adsorbent and pharmaceutical compounds play a role in their adsorption. It has been reported that π-π* interactions tend to exert a dominant influence [58]. This interaction occurs between the aromatic rings of the pharmaceutical compounds and the aromatic structure in pitch-based adsorbents. P-MPHCP showed a much better adsorption performance compared with MPHCP. These interactions are a permanent feature in aromatic compounds and gain strength as the number of aromatic rings in polymers and PPCP molecules increases. Furthermore, as shown in Figure 12, the intensities and areas of the peaks representing the π-π* interactions increased after DFS capture. Analysis of the XPS data revealed that the C1s spectrum could be sorted into five distinct peaks at approximately 284.3 (C=C), 284.8 (C–C), 285.3 (C–O), 286.4 (C=O), and 288.1 eV (π-π* interactions), respectively [59,60]. The O-atom peaks of MPHCP remained relatively consistent before and after adsorption, whereas P-MPHCP exhibited conspicuous π-π* interaction peaks post-adsorption. This observation suggests that P-MPHCP demonstrates enhanced π-π* interactions attributed to its higher benzene ring content.

Therefore, based on the analysis above, the porous structure of the adsorbent shown only provides abundant adsorption sites that, like electrostatic interactions, is not the determining reason for the adsorption capacity. π-π* interactions and hydrogen bonding are the main mechanisms of adsorption. MPHCP showed evident d-d hydrogen bonding and, after the introduction of a large amount of phenol, the P-MPHCP showed a strong Yoshida-type hydrogen bonding as well as apparent π-π* interactions.

## 3. Materials and Methods

### 3.1. Materials

Ferrosoferric oxide (Fe_3_O_4_, 50 nm particle size), anhydrous ferric chloride (FeCl_3_), 3-phenylglutaric acid, antipyrine, and dimethylformaldehyde (FDA) from Shanghai Aladdin Reagent (Shanghai, China); phenol, 1,2-dichloroethane (DCE), dichloromethane, and anhydrous methanol from Sinopharm Chemical Reagent Company (Shanghai, China); and diclofenac sodium (DFS), 4-acetylaminophenol (AMP), and sodium hydroxide (CaH_2_) from Shanghai Sahn Chemical Technology company (Shanghai, China). Before the experiment, the DCE and pitch were dried and dehydrated.

### 3.2. Preparation of Functionalized Fe_3_O_4_ Nanoparticles

To prepare the modified magnetic nanoparticles, 20 mL of deionized water and Fe_3_O_4_ nanoparticles were placed into a flask. The mixture was augmented with 3-phenylglutaric acid and subjected to heating at 80 °C with continuous mechanical stirring for 2 h. Following this, magnetic separation was employed to segregate the solid and liquid phases, with the supernatant subsequently discarded. The solid residue underwent a thorough triple wash with deionized water prior to final drying under vacuum conditions. The final product was named Fe_3_O_4_@3-phenylglutaric acid NPs. The synthesis process is shown in Figure 1.

### 3.3. Preparation of Pitch-Based HCPs

In the process of synthesizing the pitch-based HCPs, 0.608 g of pitch was introduced into 40 mL of 1,2-dichloroethane, acting as the monomer. This mixture was subjected to sonication at room temperature until complete dissolution was achieved. Under a nitrogen atmosphere, 0.912 g of dimethylformaldehyde (external crosslinker) and 2.592 g of anhydrous iron (III) chloride (Lewis acid) were introduced and stirred at 80 °C for 24 h. The resulting products were subjected to filtration through anhydrous methanol. Subsequently, further purification was carried out using a Soxhlet extractor for 12 h, employing anhydrous methanol and methylene chloride. Finally, the products underwent a 24 h drying process at 80 °C and were then designated as PHCP.

A total of 0.608 g of pitch was introduced into 40 mL of 1,2-dichloroethane and sonicated until the pitch was completely dissolved at room temperature. A total of 0.16 g of Fe_3_O_4_@3-phenylglutaric acid NPs were added to the mixture and dispersed evenly. A total of 0.912 g of dimethylformaldehyde and 2.592 g of anhydrous iron (III) chloride as a catalyzer were added to the mixture in a nitrogen atmosphere. The following steps were the same as the preparation of PHCP, the adsorbent powder obtained was named MPHCP.

To synthesize P-MPHCP, we combined 0.608 g of pitch with 40 mL of 1,2-dichloroethane along with 0.16 g of Fe_3_O_4_@3-phenylglutaric acid NPs and 0.0608 g of phenol, 0.912 g of dimethylformaldehyde, and 2.592 g of anhydrous iron(III) chloride as a Lewis acid, all in a nitrogen atmosphere. The ensuing steps closely mirrored those used for PHCP preparation, ultimately yielding the adsorbent powder known as P-MPHCP. Figure 1 shows the synthetic pathways for three pitch-based HCP adsorbents.

### 3.4. Characterizations

The methodology and equipment used for characterization, such as Fourier trans-form infrared spectroscopy (FT-IR, Platinum Elmer, USA), thermogravimetric analysis (TGA, STA449F3 Thermogravimetric instrument, NETZSCH, Germany), X-ray diffrac-tion patterns (XRD, Brock D8 ADVANCE, Brock, Switzerland), a scanning electron mi-croscope (SEM, Su8100 scanning electron microscope, Hitachi, Japan), a high-resolution transmission electron microscope (HRTEM, JEM-2100 F high-resolution transmission electron microscope, JEOL, Japan), Brunauer-Emmett-Teller analysis (BET, ASAP 2020 surface area and porosity analyzer, Micromeritics, USA), X-ray photoelectron spec-trometry (XPS, K-alpha device, Thermo Field), and Bruker 400M NMR carbon spectrum (^13^C NMR, Brock, Germany) analysis, were consistent with our previous work [30]. The structural analysis of modified magnetic nanoparticles (FT-IR, TGA, XRD, and SEM) and pitch-based adsorbents (FT-IR, TGA, XRD, SEM, TEM, VSM, and BET) were presented in detail [30].

### 3.5. Adsorption Experiments

Batch experiments were employed to investigate the adsorption of PPCPs from aqueous solutions. These experiments involved varying process parameters, including pH levels (ranging from 4 to 10), the initial concentration of PPCPs (ranging from 100 to 350 mg L^−1^), and contact time (ranging from 2 to 360 min). Equilibrium adsorption experiments were performed as follows. Pitch-based HCP adsorbents (5 mg) were administered in 5 mL of DFS, AMP, and antipyrine solutions at concentrations ranging from 100 to 350 mg L^−1^. Subsequently, the mixture was placed on a temperature-controlled shaker for 6 h to ensure equilibrium. Thereafter, the adsorbents were separated directly from the solutions through external magnets and the remaining concentrations of PPCPs were determined using a UV-VIS spectrophotometer (TU-1910, Beijing Pu-Analysis, Beijing, China). The adsorption capacity and absorbance of the adsorbent onto the PPCPs were calculated according to Equations (10) and (11) as follows:

(10) $$q_{e}=\frac{\left(C_{0}-C_{e}\right)V}{m}$$

(11) $$U=\frac{C_{0}-C_{e}}{C_{0}}\times 100\%$$

Within these parameters, *C*_0_ (mg L^−1^) signifies the initial concentration, *q_e_* (mg g^−1^) represents the equilibrium adsorption capacity, *V* (L) denotes the solution volume, *m* (g) indicates the mass of the adsorbent, *U* (%) characterizes the adsorption efficiency, and *C_e_* (mg L^−1^) stands for the equilibrium concentration of the PPCPs.

Kinetic experiments were conducted under optimal pH conditions. Each of the pitch-based HCP adsorbents (50 mg) was added to a 50 mL solution of PPCPs at a concentration of 200 mg L^−1^ at 25 °C. Solution samples were harvested at different times (2, 4, 8, 12, 20, 30, 45, 60, 120, 180, 240, and 360 min) and the residual concentration of PPCPs was quantified using UV–VIS spectrophotometry. The adsorption capacity of PPCPs onto each adsorbent at time *t* (*q_t_*) was calculated using Equation (10) where *C_t_*, the concentration at each sampling time, is substituted for *C_e_*.

The solution pH levels was modulated within the range of 4 to 10 by introducing either 0.1 M NaOH or 0.1 M HCl solution to the PPCP solution. Five mg of adsorbent was added to 5 mL of PPCP solution with different pH values (100 mg L^−1^) and adsorbed at 25 °C for 6 h. The residual concentration of PPCPs was measured to evaluate adsorption capacity.

Thermodynamic experiments were carried out at modified temperatures of 35 and 45 °C, with all other conditions remaining the same as the equilibrium experiments. After 6 h of time for the reaction, the remaining concentration of PPCPs was measured to determine the thermodynamic behavior of the adsorbents.

The performance and stability of the adsorbent were evaluated through repeated cycling experiments. The experiments were conducted at an optimal pH level and a PPCP concentration of 200 mg L^−1^. The adsorbent underwent regeneration using ethanol, which acted as a counter-extraction solution to release the adsorbed PPCPs. The residual PPCPs on the surface of the adsorbent were washed with deionized water, and the adsorbent was ultimately dried. To understand the adsorption rate of PPCPs, the desorption-treated adsorbent was exposed to another 200 mg L^−1^ PPCP solution for further adsorption.

## 4. Conclusions

Pitch-based HCP adsorbents (PHCP, M-PHCP, and P-MPHCP) were synthesized through the Friedel–Crafts alkylation method and used for the adsorption of PPCPs. For P-MPHCP, according to the Langmuir model fitting parameters, the maximum adsorption capacities of DFS, AMP, and antipyrine were 444.93, 180.89, and 223.62 mg g^−1^, respectively. Moreover, the thermodynamic study showed that the adsorption of pitch-based adsorbents onto PPCPs was a spontaneous exothermic process. Moreover, even after 5 adsorption–desorption cycles, the adsorption capacity remained nearly unchanged, demonstrating consistently high performance. This resilience was consistently maintained even throughout 10 adsorption–desorption cycles. The porous structure of the adsorbents only provides abundant adsorption sites that, like electrostatic interaction, is not the determining reason for the adsorption capacity. π-π* interactions and hydrogen bonding are the main mechanisms of adsorption. This study provides technical feasibility for the field of removing PPCPs; the low-cost pitch-based HCP has great potential practical application prospect.

## Figures and Tables

**Table 1:** Adsorption kinetics of DFS, AMP, and antipyrine onto HCPs.

Sample	PPCP	Pseudo-First Order Kinetic Model			Pseudo-Second Order Kinetic Model		
		*q_e_* (cal) (mg g^−1^)	*K*_1_ (min^−1^)	*R* ^2^	*q_e_* (cal) (mg g^−1^)	*K*_2_ (g mg^−1^ min^−1^)	*R* ^2^
PHCP	DFS	40.62	0.0045	0.5767	73.09	0.0012	0.9985
	AMP	25.64	0.0081	0.6665	54.97	0.00092	0.9959
	Antipyrine	40.58	0.0077	0.7569	81.36	0.00088	0.9993
MPHCP	DFS	106.80	0.0024	0.5128	133.51	0.00093	0.9997
	AMP	25.82	0.0060	0.6910	67.24	0.0023	0.9999
	Antipyrine	26.51	0.0095	0.8066	94.25	0.0021	0.9998
P-MPHCP	DFS	81.09	0.0050	0.6963	175.43	0.00076	0.9994
	AMP	27.13	0.011	0.9471	82.64	0.0021	0.9996
	Antipyrine	25.09	0.009	0.6517	110.01	0.0023	0.9998

**Table 2:** Correlation coefficient and isotherm parameters for the adsorption of pitch-based HCP adsorbents at 25 °C.

Sample	PPCP	Langmuir Isotherm			Freundlich Isotherm			Dubinin–Redushkevich Isotherm			
		*q_max_*	*K_L_*	*R* ^2^	*n*	*K_F_*	*R* ^2^	*q_DR_*	*B*	*E_a_*	*R* ^2^
PHCP	DFS	229.30	0.022	0.9054	2.76	28.89	0.8088	0.0028	0.0044	10.66	0.8407
	AMP	145.32	0.0038	0.9356	1.60	2.30	0.9031	0.0040	0.0084	7.72	0.9420
	Antipyrine	163.70	0.0046	0.9626	1.66	3.25	0.9322	0.0050	0.0079	7.96	0.9666
MPHCP	DFS	282.56	0.15	0.9804	4.24	95.94	0.8375	0.0026	0.0025	14.17	0.9005
	AMP	156.80	0.0058	0.9567	1.81	4.48	0.9222	0.0036	0.0070	8.47	0.8802
	Antipyrine	186.01	0.0061	0.9823	1.80	5.45	0.9573	0.0036	0.0063	8.91	0.9525
P-MPHCP	DFS	444.93	0.092	0.9116	2.30	75.06	0.8165	0.0053	0.0080	7.93	0.9195
	AMP	180.89	0.0052	0.9327	1.72	4.23	0.8958	0.0034	0.0057	9.36	0.9885
	Antipyrine	223.62	0.0058	0.9763	1.72	5.57	0.9579	0.0114	0.0044	10.64	0.8216

**Table 3:** Thermodynamic parameters for PPCP adsorption onto pitch-based HCP adsorbents.

Sample	PPCP	*T* (°C)	∆*H*° (kJ mol^−1^)	∆*S*° (J mol^−1^)	∆*G*° (kJ mol^−1^)
PHCP	DCF	25	−9.6507	−71.52	−7.7365
		35			−7.3997
		45			−6.3060
	AMP	25	−3.4053	7.12	−3.3673
		35			−4.0867
		45			−3.5096
	Antipyrine	25	−3.65246	−1.89	−3.7936
		35			−3.2094
		45			−3.7557
MPHCP	DCF	25	−12.3543	1.69	−12.543
		35			−4.9310
		45			−6.0347
	AMP	25	−2.5186	65.05	−11.753
		35			−5.3425
		45			−5.2577
	Antipyrine	25	−3.31449	45.84	−11.213
		35			−4.1104
		45			−3.7338
P-MPHCP	DCF	25	−10.1730	49.67	−12.577
		35			−6.3273
		45			−4.1057
	AMP	25	−4.3284	30.98	−12.914
		35			−5.6500
		45			−4.3574
	Antipyrine	25	−5.2217	4.31	−12.747
		35			−5.7737
		45			−5.2127

## Data Availability

Data are contained within the article and Supplementary Materials.

## Supplementary Materials

The following supporting information can be downloaded at: https://www.mdpi.com/article/10.3390/molecules29020463/s1. Figure S1: Standard curves of DFS (a,d), AMP (b,e), and antipyrine (c,f). Figure S2: Isotherm adsorption capacity for pitch-based HCP adsorbents onto PPCPs (a) DFS, (b) AMP, and (c) antipyrine with different initial concentrations at 35 °C. Figure S3: Isotherm adsorption capacity for pitch-based HCP adsorbents onto PPCPs (a) DFS, (b) AMP, and (c) antipyrine with different initial concentrations at 45 °C. Figure S4: Langmuir (a–c), Freundlich (d–f), and D–R (g–i) isotherms for the adsorption of pitch-based HCP adsorbents onto DFS, AMP, and antipyrine at 35 °C. Figure S5: Langmuir (a–c), Freundlich (d–f), and D–R (g–i) isotherms for the adsorption of pitch-based HCP adsorbents onto DFS, AMP, and antipyrine at 45 °C. Table S1: Correlation coefficient and isotherm parameters for the adsorption of pitch-based HCP adsorbents at 35 °C. Table S2: Correlation coefficient and isotherm parameters for the adsorption of pitch-based HCP adsorbents at 45 °C. Table S3: DFS adsorption performance of P-MPHCP compared with other reported adsorbents. Figure S6: Chemical and structural characteristics of three PPCPs. References [22,32,45,46,47,48,52,53,61,62,63,64] are cited in the supplementary materials.
