# Supplementary material for: Elimination of Pharmaceutical Compounds from Aqueous Solution through Novel Functionalized Pitch-Based Porous Adsorbents: Kinetic, Isotherm, Thermodynamic Studies and Mechanism Analysis
Source: Molecules. 2024 Jan 17;29(2):463. doi: 10.3390/molecules29020463 (PMC10819009; doi:10.3390/molecules29020463)
Supplement: Supplementary file 1 [file molecules-29-00463-s001.zip › molecules-2829425-supplementary.pdf]

Supplementary Information for

Elimination of pharmaceutical compounds from aqueous solution by novel functionalized pitch-based porous adsorbents: Kinetic, isotherm, thermodynamic studies and mechanism analysis

Qilin Yang[1], Hongwei Zhao [1,2,*], Qi Peng[1], Guang Chen[1], Jiali Liu[1], Xinxiu Cao[1,2], Shaohui Xiong[1,2], Gen Li[1,2], Qingquan Liu [1,2,*]

[1] *School of Material Science and Engineering, Hunan University of Science and Technology, Xiangtan 411201, China*

[2] *Hunan Provincial Key Laboratory of Advanced Materials for New Energy Storage and Conversion, Hunan University of Science and Technology, Xiangtan 411201, China*

* *Corresponding Author: Hongwei Zhao, E-mail: hwzhao@hnust.edu.cn; Qingquan Liu, E-mail: qqliu@hnust.edu.cn.*

Table of Contents

**Figure S1**	Standard curves of DFS(a,d), AMP(b,e), and Antipyrine(c,f).
**Figure S2**	Isotherm adsorption capacity for pitch-based HCP adsorbents toward PPCPs (a) DFS, (b) AMP, and (c) Antipyrine with different initial concentrations at 35 °C.
**Figure S3**	Isotherm adsorption capacity for pitch-based HCP adsorbents toward PPCPs (a) DFS, (b) AMP, and (c) Antipyrine with different initial concentrations at 45 °C.
**Figure S4**	Langmuir (a-c), Freundlich (d-f), and D-R (g-i) isotherms for the adsorption of pitch-based HCP adsorbents toward DFS, AMP, and antipyrine at 35 °C.
**Figure S5**	Langmuir (a-c), Freundlich (d-f), and D-R (g-i) isotherms for the adsorption of pitch-based HCP adsorbents toward DFS, AMP, and antipyrine at 45 °C.
**Table S1**	Correlation coefficient and isotherm parameters for the adsorption of pitch-based HCP adsorbents at 35 °C.
**Table S2**	Correlation coefficient and isotherm parameters for the adsorption of pitch-based HCP adsorbents at 45 °C.
**Table S3**	DFS adsorption performance of P-MPHCP compared to other reported adsorbents.
**Figure S6**	Chemical and structural characteristics of three PPCPs.

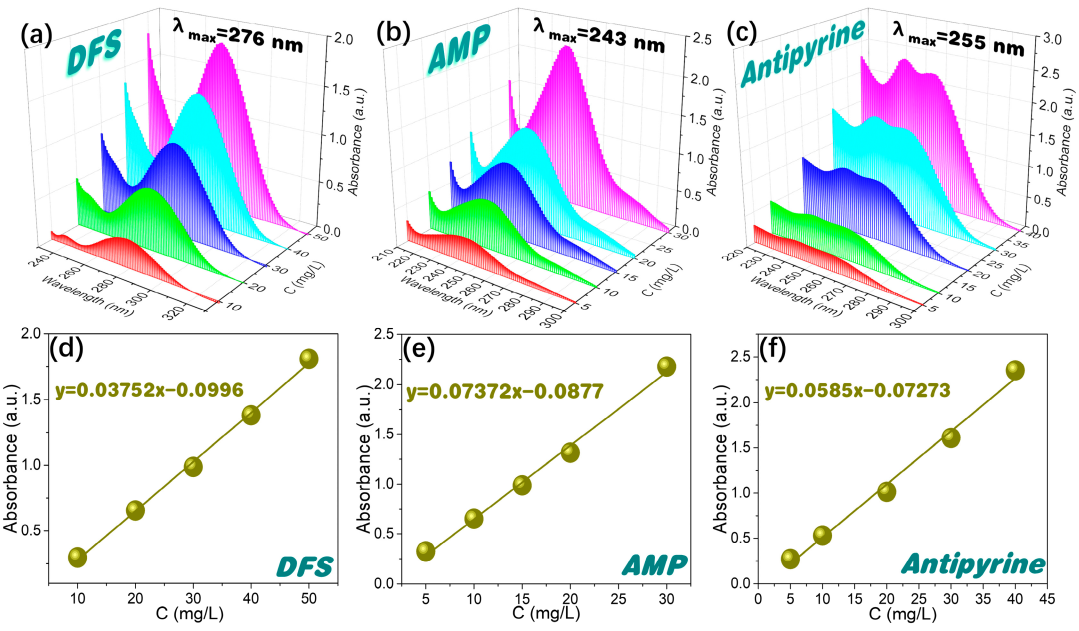

Fig. S1. Standard curves of DFS(a,d), AMP(b,e), and Antipyrine(c,f).

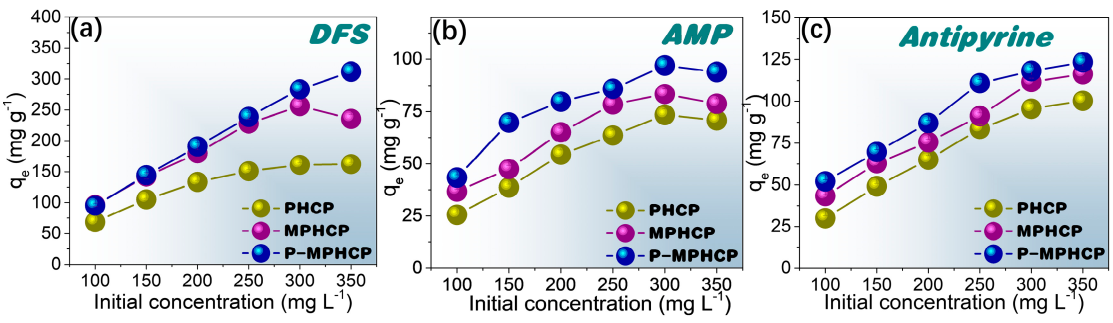

Fig. S2. Isotherm adsorption capacity for pitch-based HCP adsorbents toward PPCPs (a) DFS, (b) AMP, and (c) Antipyrine with different initial concentrations at 35 °C.

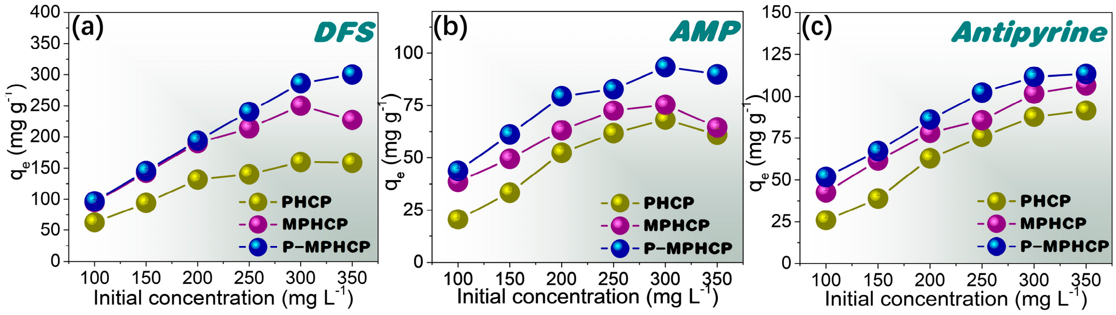

Fig. S3. Isotherm adsorption capacity for pitch-based HCP adsorbents toward PPCPs (a) DFS, (b) AMP, and (c) Antipyrine with different initial concentrations at 45 °C.

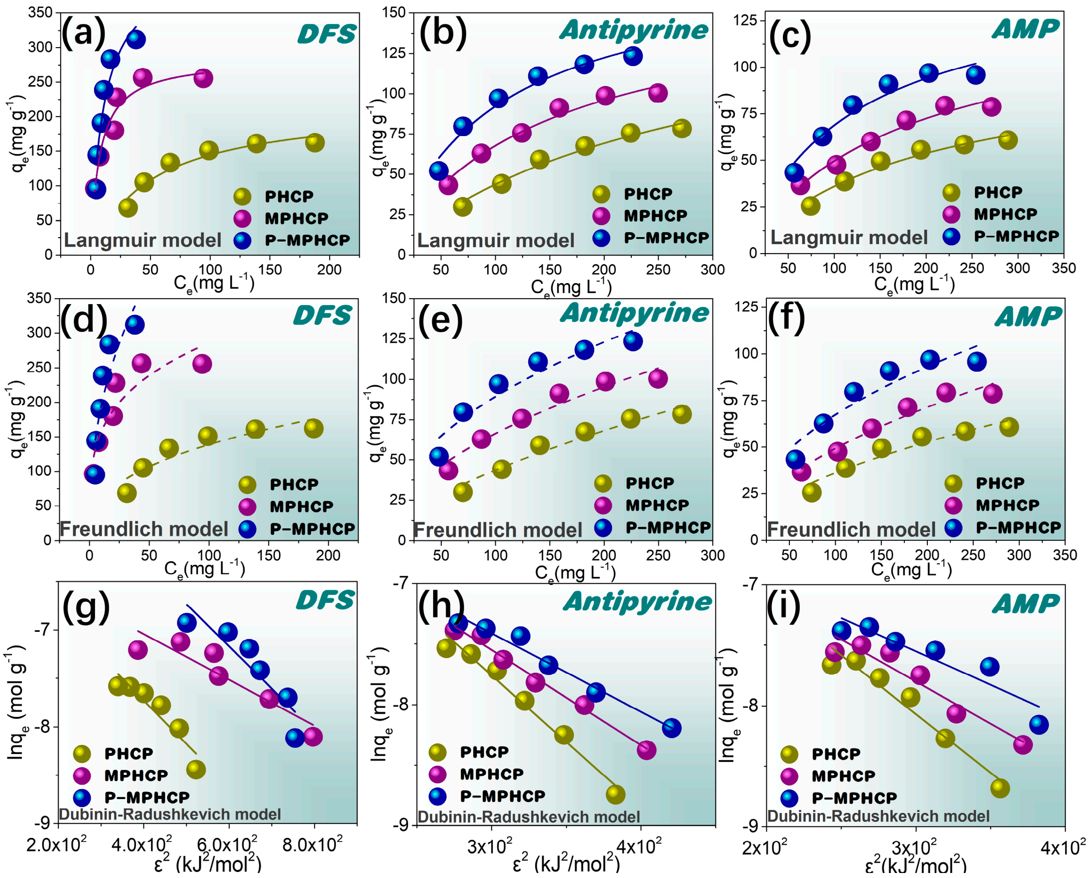

Fig. S4. Langmuir (a-c), Freundlich (d-f), and D-R (g-i) isotherms for the adsorption of pitch-based HCP adsorbents toward DFS, AMP, and antipyrine at 35 °C.

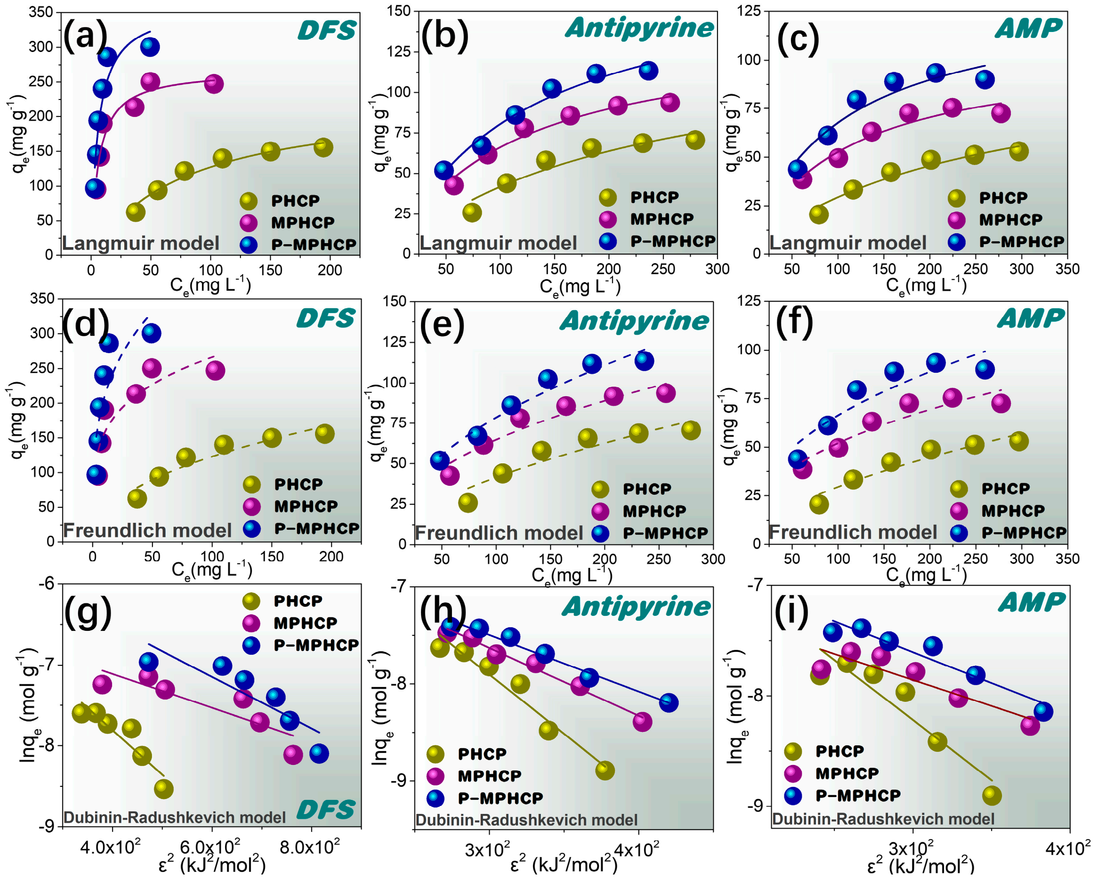

Fig. S5. Langmuir (a-c), Freundlich (d-f), and D-R (g-i) isotherms for the adsorption of pitch-based HCP adsorbents toward DFS, AMP, and antipyrine at 45 °C.

Table S1. Correlation coefficient and isotherm parameters for the adsorption of pitch-based HCP adsorbents at 35 °C.

Sample	PPCP	Langmuir isotherm			Freundlich isotherm			Dubinin-Redushkevich isotherm			
		q_{max}	K_L	R^2	n	K_F	R^2	q_{DR}	B	E_a	R^2
PHCP	DFS	217.3	0.0198	0.9339	2.658	24.55	0.8512	0.0027	0.0045	10.55	0.8697
	AMP	105.4	0.0052	0.9595	1.828	2.908	0.9223	0.0057	0.0097	7.19	0.9495
	Antipyrine	163.9	0.0037	0.9757	1.555	2.260	0.9538	0.0115	0.0110	6.75	0.9751
MPHCP	DFS	283.8	0.1329	0.9418	3.888	87.38	0.8454	0.0022	0.0024	14.56	0.8475
	AMP	134.8	0.00581	0.9713	1.863	4.159	0.9513	0.0033	0.0069	8.49	0.9172
	Antipyrine	165.1	0.0069	0.9768	1.919	6.025	0.9455	0.0055	0.0079	7.98	0.9876
P-MPHCP	DFS	424.0	0.1062	0.9142	2.909	82.57	0.8080	0.0106	0.0044	10.70	0.8317
	AMP	155.1	0.0064	0.9734	1.878	5.126	0.9414	0.0027	0.0055	9.53	0.8630
	Antipyrine	210.3	0.0059	0.9860	1.738	5.404	0.9632	0.0042	0.0065	8.79	0.9726

Table S2. Correlation coefficient and isotherm parameters for the adsorption of pitch-based HCP adsorbents at 45 °C.

Sample	PPCP	Langmuir isotherm			Freundlich isotherm			Dubinin-Redushkevich isotherm			
		q_{max}	K_L	R^2	n	K_F	R^2	q_{DR}	B	E_a	R^2
PHCP	DFS	227.7	0.0127	0.9573	2.2011	15.18	0.9004	0.0036	0.00548	9.55	0.8536
	AMP	100.9	0.0041	0.9476	1.6811	1.915	0.9126	0.0071	0.0109	6.77	0.8714
	Antipyrine	133.7	0.0046	0.9125	1.6978	2.766	0.8699	0.0143	0.0122	6.40	0.9539
MPHCP	DFS	267.7	0.1597	0.9217	4.3657	92.74	0.8149	0.0019	0.0021	15.43	0.7543
	AMP	106.2	0.0098	0.9317	2.4176	7.748	0.8778	0.0016	0.00475	10.26	0.8090
	Antipyrine	139.8	0.0090	0.9652	2.1889	7.904	0.9168	0.0040	0.007	8.45	0.9826
P-MPHCP	DFS	361.1	0.1711	0.9469	4.0110	95.21	0.8232	0.0053	0.00319	12.52	0.7770
	AMP	133.8	0.0102	0.9198	2.3731	8.149	0.8708	0.0027	0.00561	9.44	0.9182
	Antipyrine	196.0	0.0058	0.9767	1.7488	5.073	0.9486	0.0032	0.00582	9.27	0.9748

Table S3. DFS adsorption performance of P-MPHCP compared to other reported adsorbents.

Adsorbent	pH	q_{max} (mg/g) (Langmuir model)	Stabilization time (h)	Reference
P-MPHCP	5	444	1	This work
Expanded Graphite	-	330	-	[1]
OAC(2.0)	4.2	487	3	[2]
Commercial AC	4.2	76	5	[2]
PCDM-1000	-	400	12	[3]
ZIF-8	-	100	12	[3]
OH-MCOF	5	203	0.5	[4]
CTAB-ZIF-67	8	43.9	1	[5]
P-POP-1	4	166	2	[6]
P-POP-2	4	217	1	[6]
PONF	4	380.8	0.5	[7]
ZCPC-10	7.4	70	-	[8]
ZCPC-20	7.4	134	-	[8]
ZCPC-30	7.4	159	-	[8]
CNT/Al_2O_3	4	27	-	[9]
Fe_3O_4@MOF-100(Fe)	5	377	2	[10]

.

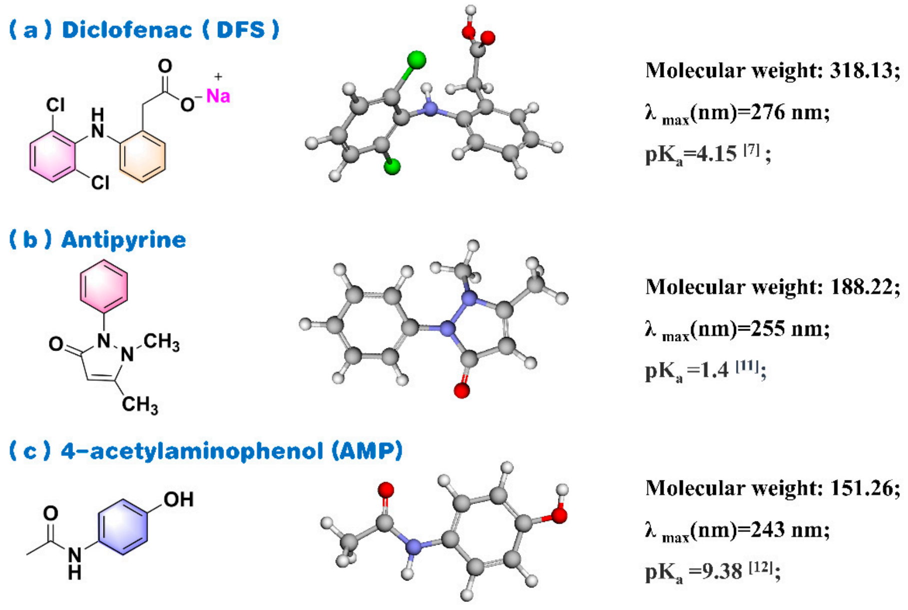

Fig. S6. Chemical and structural characteristics of three PPCPs [7, 11, 12].

References

[1] Vedenyapina M.D., Borisova D.A., Simakova A.P., et al. Adsorption of diclofenac sodium from aqueous solutions on expanded graphite[J]. Solid Fuel Chemistry, 2013, 47(1): 59-63.

[2] Bhadra B.N., Seo P.W., Jhung S.H. Adsorption of diclofenac sodium from water using oxidized activated carbon[J]. Chemical Engineering Journal, 2016, 301: 27-34.

[3] Bhadra B.N., Ahmed I., Kim S., et al. Adsorptive removal of ibuprofen and diclofenac from water using metal-organic framework-derived porous carbon[J]. Chemical Engineering Journal, 2017, 314: 50-58.

[4] Mi X., Zhou S., Zhou Z., et al. Adsorptive removal of diclofenac sodium from aqueous solution by magnetic COF: Role of hydroxyl group on COF[J]. Colloids and Surfaces A: Physicochemical and Engineering Aspects, 2020, 603: 125238.

[5] Andrew Lin K.Y., Yang H., Lee W.D. Enhanced removal of diclofenac from water using a zeolitic imidazole framework functionalized with cetyltrimethylammonium bromide (CTAB)[J]. RSC Advances, 2015, 5(99): 81330-81340.

[6] Ravi S., Choi Y., Choe J.K. Novel phenyl-phosphate-based porous organic polymers for removal of pharmaceutical contaminants in water[J]. Chemical Engineering Journal, 2020, 379: 122290.

[7] Ravi S., Choi Y., Wu S., et al. Porous organic nanofiber polymers as superfast adsorbents for capturing pharmaceutical contaminants from water[J]. Environmental Science: Nano, 2022, 9(2): 730-741.

[8] Krajisnik D., Dakovic A., Milojevic M., et al. Properties of diclofenac sodium sorption onto natural zeolite modified with cetylpyridinium chloride[J]. Colloids and Surfaces B: Biointerfaces, 2011, 83(1): 165-172.

[9] Wei H., Deng S., Huang Q., et al. Regenerable granular carbon nanotubes/alumina hybrid adsorbents for diclofenac sodium and carbamazepine removal from aqueous solution[J]. water research, 2013, 47(12): 4139-4147.

[10] Zheng X., Wang J., Xue X., et al. Facile synthesis of Fe_3O_4@MOF-100(Fe) magnetic microspheres for the adsorption of diclofenac sodium in aqueous solution[J]. Environmental Science and Pollution Research, 2018, 25(31): 31705-31717.

[11] Sangster J .Phase Diagrams and Thermodynamic Properties of Binary Organic

Systems Based on 1,2-, 1,3-, 1,4-Diaminobenzene or Benzidine[J].Journal of Physical & Chemical Reference Data, 1994, 23(2):295-338.

[12] Mark F. H. and Jennifer L. L. Determination of log$_{Kow}$ Values for Four Drugs. Journal of Chemical Education 2014 91(6),915-918.
